# Equine Mesenchymal Stem/Stromal Cells Freeze-Dried Secretome (Lyosecretome) for the Treatment of Musculoskeletal Diseases: Production Process Validation and Batch Release Test for Clinical Use

**DOI:** 10.3390/ph14060553

**Published:** 2021-06-10

**Authors:** Michela Mocchi, Stefano Grolli, Silvia Dotti, Dario Di Silvestre, Riccardo Villa, Priscilla Berni, Virna Conti, Giulia Passignani, Francesca Brambilla, Maurizio Del Bue, Laura Catenacci, Milena Sorrenti, Lorena Segale, Elia Bari, Pierluigi Mauri, Maria Luisa Torre, Sara Perteghella

**Affiliations:** 1 Department of Drug Sciences, University of Pavia, Viale Taramelli 12, 27100 Pavia, Italy; michela.mocchi@unipv.it (M.M.); laura.catenacci@unipv.it (L.C.); milena.sorrenti@unipv.it (M.S.); elia.bari@unipv.it (E.B.); sara.perteghella@unipv.it (S.P.); 2 Department of Veterinary Medical Science, University of Parma, 43121 Parma, Italy; stefano.grolli@unipr.it (S.G.); priscilla.berni@unipr.it (P.B.); virna.conti@unipr.it (V.C.); 3 Istituto Zooprofilattico Sperimentale della Lombardia e dell’Emilia Romagna, 25124 Brescia, Italy; silvia.dotti@izsler.it (S.D.); riccardo.villa@izsler.it (R.V.); 4 Institute of Biomedical Technologies, F.lli Cervi 93, 20054 Segrate, Italy; dario.disilvestre@itb.cnr.it (D.D.S.); giulia.passignani@itb.cnr.it (G.P.); francesca.brambilla@itb.cnr.it (F.B.); pierluigi.mauri@itb.cnr.it (P.M.); 5 Freelance Veterinary Medical Doctor, 43121 Parma, Italy; mauriziodelbue@gmail.com; 6 Department of Pharmaceutical Sciences, University of Piemonte Orientale, Largo Donegani 2/3, 28100 Novara, Italy; lorena.segale@uniupo.it; 7 PharmaExceed S.r.l., Piazza Castello, 19, 27100 Pavia, Italy

**Keywords:** mesenchymal stem cells, secretome, regenerative medicine, musculoskeletal disorders, equine

## Abstract

In the last decades, it has been demonstrated that the regenerative therapeutic efficacy of mesenchymal stromal cells is primarily due to the secretion of soluble factors and extracellular vesicles, collectively known as secretome. In this context, our work described the preparation and characterization of a freeze-dried secretome (Lyosecretome) from adipose tissue-derived mesenchymal stromal cells for the therapy of equine musculoskeletal disorder. An intraarticular injectable pharmaceutical powder has been formulated, and the technological process has been validated in an authorized facility for veterinary clinical-use medicinal production. Critical parameters for quality control and batch release have been identified regarding (i) physicochemical properties; (ii) extracellular vesicle morphology, size distribution, and surface biomarker; (iii) protein and lipid content; (iv) requirements for injectable pharmaceutical dosage forms such as sterility, bacterial endotoxin, and Mycoplasma; and (v) in vitro potency tests, as anti-elastase activity and proliferative activity on musculoskeletal cell lines (tenocytes and chondrocytes) and mesenchymal stromal cells. Finally, proteins putatively responsible for the biological effects have been identified by Lyosecretome proteomic investigation: IL10RA, MXRA5, RARRES2, and ANXA1 modulate the inflammatory process RARRES2, NOD1, SERPINE1, and SERPINB9 with antibacterial activity. The work provides a proof-of-concept for the manufacturing of clinical-grade equine freeze-dried secretome, and prototypes are now available for safety and efficacy clinical trials in the treatment of equine musculoskeletal diseases

## 1. Introduction

Musculoskeletal disorders (MSDs) have an extensive and growing impact representing a major health problem worldwide. Despite years of research, the understanding of these diseases and the control on their progression has not yet been achieved; indeed, different therapeutic approaches are being investigated to prevent the disorders or to promote recovery and/or regeneration of the musculoskeletal system, including muscles, bones, and joints [1,2]. MSDs often result from traumatic injuries due, for example, to vigorous physical activity leading to excessive muscle fatigue, damage of joint structures (articular cartilage, subchondral bone, synovial membrane), and inflammation or lesion of the tendon/ligament structure. Structural and genetic factors are also associated with musculoskeletal diseases onset and progression [3]. MSDs represent a high percentage of orthopedic clinical pathologies in veterinary practice and, mainly, in equine practice. Due to the low healing capacity of tissues involved in joint and tendon disorders, there is a tendency to develop chronic diseases with a significant clinical relevance [4]. Beyond the traditional treatments (local or systemic nonsteroidal anti-inflammatory drugs, intralesional steroids, correct shoeing, chondroprotectants such as hyaluronic acid), innovative regenerative therapies, including the use of Mesenchymal Stem/Stromal Cells (MSCs), have emerged over the past ten years as promising agents to promote local repair. Especially in the veterinary field, many clinical applications of cell-based therapies have been described, starting from the early 2000s with the first application of MSCs on the equine model [5,6]. Despite the considerable amount of data suggesting the safety and efficacy of MSCs in experimental animal models and preclinical studies, cell-based therapies are not yet routinely applied in the clinic [7,8,9,10]. Over the years, the therapeutic application of MSCs has known a paradigm shift: Nowadays, their engraftment, proliferation, and differentiation properties are not considered key features of their therapeutic abilities. Instead, the main actors of MSCs benefit are now considered several bioactive molecules (including proteins, cytokines, chemokines, growth factors, but also nucleic acid) released by cells and involved in cell-to-cell communication and cross-talk [11]. Known as secretome, this complex set of secreted factors and vesicles seems to represent a valid alternative as a cell-free therapy, offering several advantages in comparison to cell-based therapy, such as lower immunogenicity, ease of storage and handling, and lower cost ensuring usefulness and feasibility in the clinic [12,13]. However, addressing a suitable medicinal product is challenging, and further investigations are needed to shed light on secretome composition, properties, and in vivo behavior. Indeed, while evaluating secretome safety and efficacy for clinical use, many aspects, ranging from production processes to molecular characterization, must be defined.

In this study, an injectable freeze-dried pharmaceutical powder containing equine secretome has been formulated (Lyosecretome); the technological process has been validated in an authorized facility for veterinary clinical-grade medicinal production. Critical parameters for quality control and batch release have been identified regarding (i) physicochemical powder properties; (ii) extracellular vesicle morphology, size distribution, and surface biomarkers; (iii) protein and lipid content; (iv) requirements for injectable pharmaceutical dosage forms (sterility, bacterial endotoxin, and Mycoplasma); and (v) in vitro potency tests, and efficacy on proliferative induction of selected cell lines. Finally, putative proteins responsible for the biological effects have been identified by a Lyosecretome proteomic investigation based on nano Liquid Chromatography coupled with high-resolution mass spectrometry.

## 2. Results

### 2.1. Lyosecretome Production

Three different equine AD-MSCs lines from the biobank meet all the requirements for clinical use as all the steps were made according to ISO 9001:2018 clinical grade. Secretome production was induced by 24 h serum starvation; after this time, the supernatants were collected, and cells were detached to estimate their total amount and viability (Table 1).

The supernatants were first centrifuged to eliminate cell debris, followed by an ultrafiltration process to concentrate and filter. The concentration step allows to reduce the volume, reaching a fixed value of cell equivalents per mL (0.5 × 10^6^ cell/mL); instead, the filtration phase with 5 kDa molecular weight cut-off allows one to retain both EVs and soluble proteins contained in the supernatants. At the end of the concentration step, the obtained volume was diafiltered at least five times with ultrapure deionized water to erase any tracks of undesired compounds. The starting volume for all the produced batches was about 800 mL, the time process lasted approximately 3 h using a filtration module with a superficial area of 370 cm^2^, and the calculated ultrafiltration efficiency was 17.8 ± 2.3 L/m^2^/h (mean value ± standard deviation, *n* = 3). Once the purified solution was obtained, mannitol was added as the selected cryoprotectant. Samples were stored at −80 °C before the freeze-drying process. In this timeframe, to ensure the quality of the product, microbiological tests were performed during all the production phases to prove that the sterility conditions were maintained during the formulation along with the final product. Microbiological tests were conducted by an accredited and certified facility (IZSLER, Brescia, Italy), which routinely controls cell and cell-derived products for veterinary clinical use. These tests affirmed: (i) The absence of any mycoplasma and (ii) bacterial endotoxin level in an acceptable range (Table 2).

### 2.2. Lyosecretome Characterization

After the lyophilization process, a technological characterization was performed on Lyosecretome powder.

To check the suitability of each formulation for a future in vivo administration, further analyses were performed on the final product. For each formulation, cake’s appearance was evaluated by randomly selecting four vials and performing a visual inspection. Following the aqueous solvent’s evaporation during lyophilization, a solid and porous cake was formed, free of defects. The lyophilized secretome dissolved rapidly by a gentle agitation adding water, generating a clear suspension free of visible particles. The osmolarity was always within the acceptable range for injectable formulations (250–350 mOsm/Kg) and always between 320 and 350 mOsm/Kg. The pH value was always in the range of 7.2 to 7.6.

**Figure 1:**
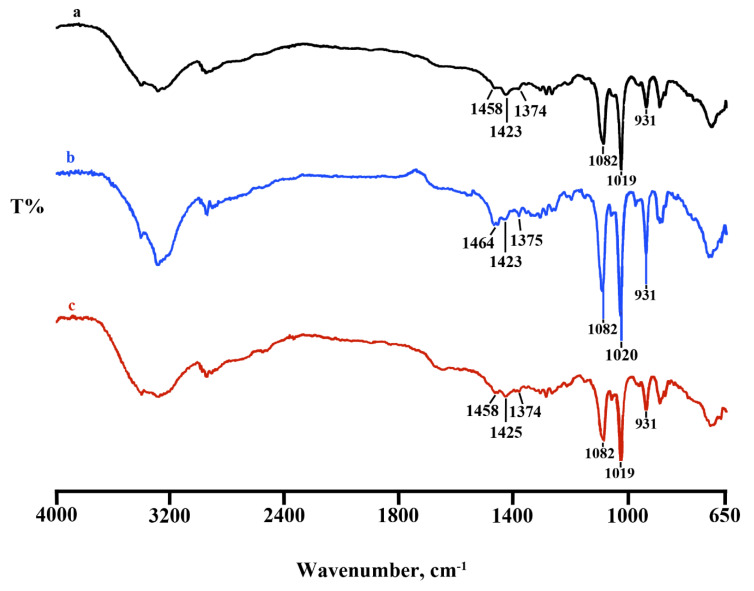
Comparison between FTIR spectra of Lyosecretome batch 1 (spectrum a), 2 (spectrum b), and 3 (spectrum c), respectively.

FTIR spectra were performed on Lyosecretome batches. Comparing the three batches reported in Figure 1, the spectral region goes from 650 to 4000 cm^−1^; the batches, as expected, have similar bands at around at 1082 cm^−1^ and 1019 cm^−1^ and 930 cm^−1^; those picks are predominant, and unfortunately, in this region, there is an overlap with the characteristic mannitol vibrations bands; thus, it is difficult to distinguish the Lyosecretome presence (Figure 1). Lyosecretome bands were also detected in the spectral region between 1423 cm^−1^ and 1458 cm^−1^, pointing out the presence of CH_2_ groups and 1374 cm^−1^ and 1375 cm^−1^ related to CH_3_ groups, thus confirming the presence of lipids and protein.

**Figure 2:**
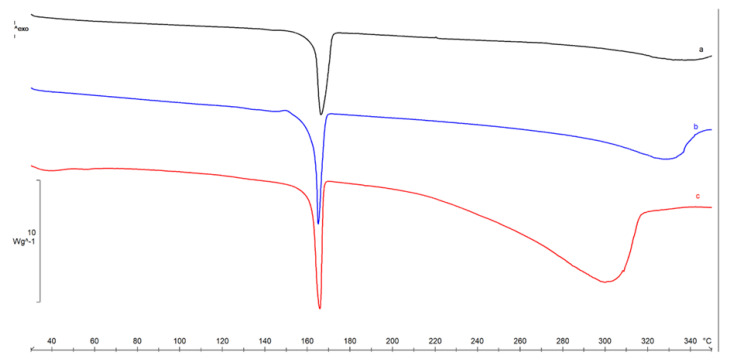
DSC profiles of Lyosecretome batches 1, 2, and 3 (curves a, b, and c).

The three batches were analyzed by DSC, and the thermal profile was reported below as a comparison (Figure 2). The endothermic peak at 164.3 ± 0.8 °C (ΔH = 278 ± 3 Jg^−1^) was due to the melting of crystalline mannitol (*β* form), followed by sample decomposition.

**Figure 3:**
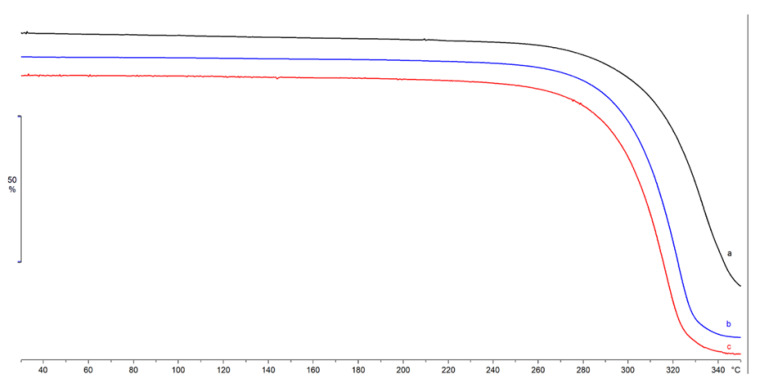
TGA profiles of Lyosecretome batches 1, 2, and 3 (curves a, b, and c).

The anhydrous nature of the lyophilized samples was confirmed by TGA analysis (Figure 3), where no mass losses were recorded in the temperature range 50–100 °C, related to residual water. An almost complete mass loss was recorded for all the samples starting from about 244 °C due to sample decomposition, confirming the same thermal stability for the three batches.

Morphological investigation through SEM images showed that vesicles integrity and spherical shape were maintained for all three batches, demonstrating that ultrafiltration and lyophilization processes did not influence particle structure (data not presented).

**Figure 4:**
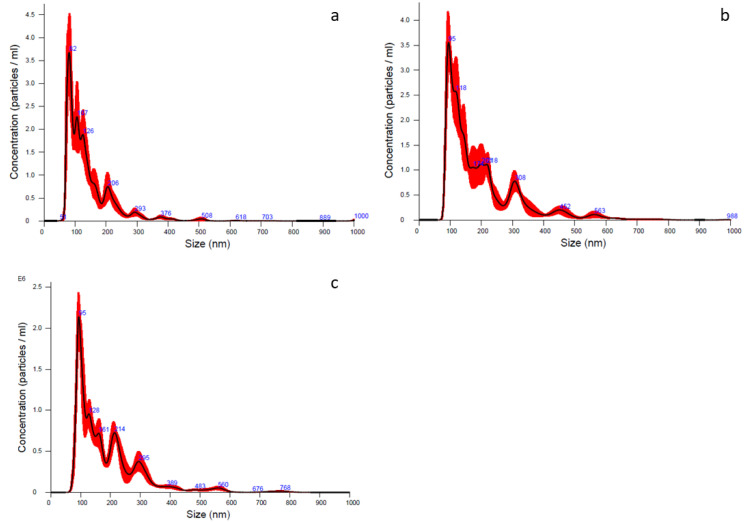
NTA analyses showing averaged particles concentration and size distribution of batch 1 (a), 2 (b), and 3 (c), respectively. The average of each batch was made by processing 6 cycles of 60 s each. The red part indicates the ± standard error of the mean values (*n* = 3). Particles concentration is expressed as mean value ± standard deviation (*n* = 3).

Particle size characterization has been performed on Lyosecretome using NTA technology to (i) count nanoparticles observed in a specific volume, (ii) measure the diameter, and (iii) obtain the particle concentration. The analysis detected a heterogeneous population including nano/micro-structured vesicles; in detail, for all three batches, 10% of the population diameter (d_10_) ranged 77–95 nm, 50% of the population diameter (d_50_) ranged 115–156 nm, and 90% of the population diameter (d_90_) was less than 260 nm. Regarding particle concentration, it ranged between 1.77 × 10^8^ and 3.47 × 10^8^ particle/mL based on the considered batches (Table 3 and Figure 4).

**Figure 5:**
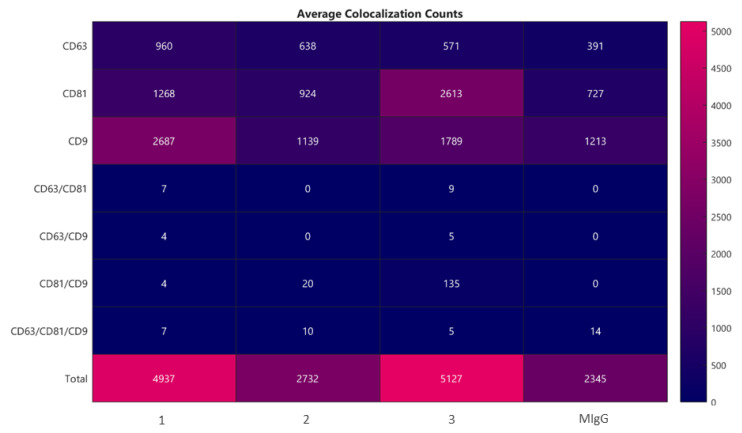
Average colocalization counts evaluating the correlations between proteins expressed on exosome’s surfaces and fluorescent antibodies markers CD63, CD81, and CD9. According to the average results of the three determinations (without considering the negative control murine IgG), it is possible to underline that there were 723 exosomes positive only for the anti-CD-63 human antibody, approximately 1601 exosomes positive only for the anti-CD-81 human antibody, 1871 exosomes positive only for the anti-CD-9 human antibody, 5 exosomes have a double positive match for anti-CD-81 and anti-CD-63 human antibodies, approximately 5 exosomes have a double positive match for anti-CD-63 and anti-CD-9 human antibodies, 53 exosomes are simultaneously positive for anti-CD-9 and anti-CD-81 human antibodies, and 7 exosomes present a triple positive match for human antibodies.

The number and size of exosomes were determined by ExoView with biomarker detection. This method is based on a direct-biomarker association that captures particles into a chip. ExoView also allows discriminating with fluorescence if a protein is expressed on the surface or into the exosome. The analysis method could be divided into two macro steps: Interferometric imaging that gives information regarding the exosome’s number and size, and fluorescence analysis that gives the intensity of a positive-marker protein. The data accuracy depends on correct binding between proteins expressed by exosomes and extracellular vesicles and their specific antibodies marker. As a result, a first consideration was a correlation between the horse’s protein and human protein: Data confirm that human capture antibodies had a fixed animal ligand. Moreover, the average colocalization counts are shown in Figure 5 (triplicate analysis). The correlations between proteins expressed on exosome’s surfaces and fluorescent antibody markers can be vertically read in the chart. The last plot column indicates the negative control performed by Murine IgG. In the first plot, column data show that in a horse’s secretome, there is a high number of exosome’s surface proteins responsive to the anti-CD-9 fluorescent human antibody, and the second most-expressed ligand is a protein that could be correlated to anti-CD-81 fluorescent human antibody. Finally, the lower capture marker is the anti-CD-63 fluorescent human antibody. There is also double or triple positive binding between the surface protein and human antibodies, as the seven exosomes are simultaneously expressed on the external membrane proteins positive to anti-CD-81 and anti-CD-63 fluorescent human antibodies.

Lyosecretome was characterized in terms of total lipid and protein content, and results are reported in Table 4 expressed as μg of protein or lipid per mg of Lyosecretome.

Focusing on the yield of each batch, for batch 2, we obtained a higher amount of both protein and lipid content, and comparing the three batches overall, a significant difference was found (*p* < 0.05). Moreover, it is evident that there was a correlation between protein and lipid content. It is well known that cell lines can highly affect secretome production and composition due to the variability within the same species. This aspect could explain the results in terms of different yield, although a certain reproducibility is maintained.

It should be noticed that the methods applied to analyze protein and lipid content meet the pharmaceutical conditions and were previously validated [14]. For BCA-protein quantification, the mean coefficient of calibration curve determination was in a range of 0 up to 100 μg/mL of BSA in water; the curve equation has an intercept not statically significant, and the plot of the residuals had a common distribution of the error. These latter considerations mean that no systematic error influenced the calibration (data not shown). Considering the lipid quantification that was performed by a Nile Red assay, the mean coefficient of the calibration curve determination was within a range of 0–20 μg/mL of PC in water; the distribution of the plot residuals had a normal trend of the error; thus, no systematic error influenced the calibration (data not shown).

### 2.3. In Vitro Potency Test

**Figure 6:**
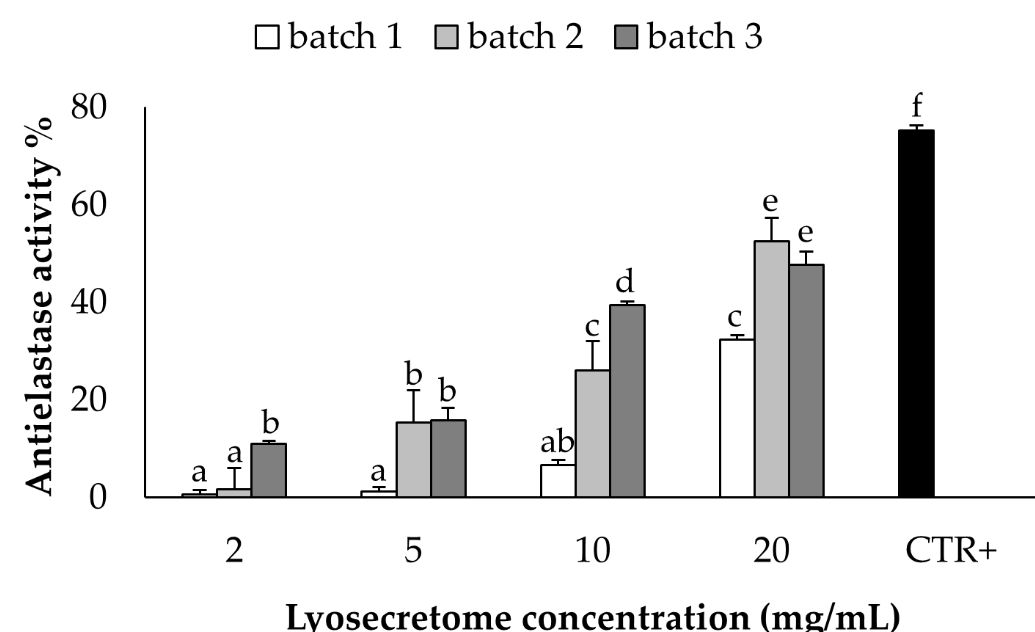
Anti-elastase activity was tested for the three batches at different Lyosecretome concentrations: 2, 5, 10, and 20 mg/mL, obtaining a dose-dependent trend. Mean values ± LSD (*n* = 3), ANOVA. Different letters (a, b, c, d, e, and f) indicate significant differences between the means (*p* < 0.05), whereas the same letter indicates no significant difference (*p* > 0.05).

The anti-elastase activity was tested on equine Lyosecretome at different concentrations (2, 5, 10, and 20 mg/mL), showing a dose-dependent trend (Figure 6). The activity assay consists of a kinetics enzymatic reaction considered from time 0 up to 40 min. The activity exhibited almost 50% at the higher concentration, considering Epigallocatechin gallate as a positive control due to its positive inhibition of the elastase enzyme.

**Figure 7:**
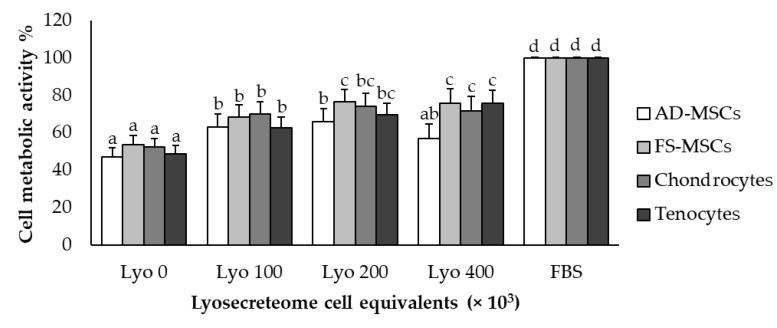
Cell metabolic activity on AD-MSCs, SF-MSCs, tenocytes, and chondrocytes tested at different Lyosecretome concentrations demonstrating a dose-dependency up to 200,000 cell equivalents/well. Mean values ± LSD (*n* = 4), ANOVA. Different letters (a, b, c, d) indicate significant differences between the means (*p* < 0.05), whereas the same letter indicates no significant difference (*p* > 0.05). FBS indicates the positive control, while Lyo 0 stands for the negative control.

The metabolic activity of equine AD-MSCs, SF-MSCs, tenocytes, and chondrocytes was evaluated after treatment with Lyosecretome. In detail, Lyosecretome was tested at the concentrations of 400,000, 200,000, and 100,000 cell equivalents/well. For all cell types, the exposition to Lyosecretome stimulated cell proliferation compared to serum-free medium (*p* < 0.0001), and a dose-dependent trend of cell metabolic activity was observed. The effects of Lyosecretome treatment were statistically different in the four cell types (*p* < 0.0001). As shown in the figure below (Figure 7), the dose-dependency reached a plateau at 200,000 cell equivalent/well. The medium with 10% FBS added was considered a positive control, while the serum-free medium was used as a negative control.

### 2.4. Proteomic Investigation

**Figure 8:**
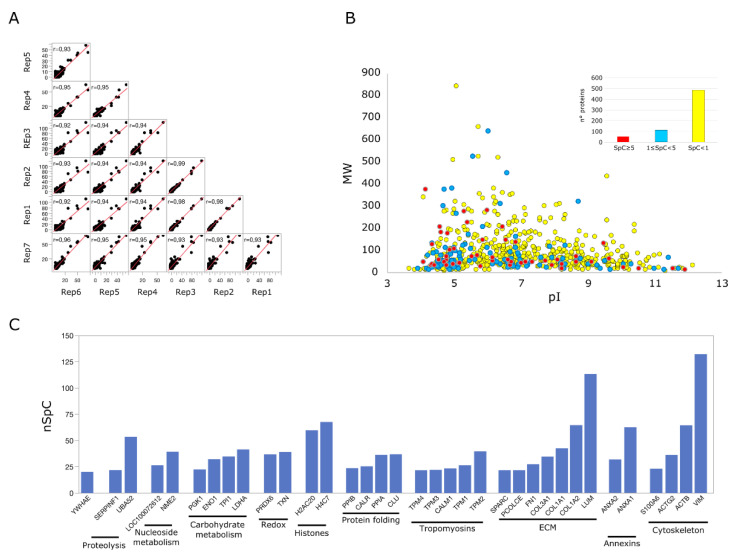
Proteomic analysis secreted from equine mesenchymal stem cells. (**A**) Spearman’s correlation computed using Spectral counts of replicate LC-MS/MS analysis. (**B**) Virtual 2D-Map (pI vs. MW) of the total proteins identified (*n* = 647); color codes indicated the average spectral counts characterizing the identified proteins. (**C**) Rank list of the most abundant identified proteins (*n* = 35, nSpC > 20) clustered for functional categories.

The protein characterization of the secretome (Lyosecretome) isolated from equine mesenchymal stem cells allowed the identification of 647 distinct proteins (Table S1). This proteome was defined by seven technical replicate analyses performed with two different mass spectrometers. Regardless of the instrumentation used, all replicates showed good repeatability with R^2^ values ranging from 0.92 to 0.99 (Figure 8A). About 25% of the proteins were identified by an average spectral count > 1 (Figure 8B). In addition, 95 proteins were identified in all analyzed samples, while 272 were found in at least 2 replicate analyses out of 7 (Table S1).

The rank list of the most abundant identified proteins (Figure 8C) highlighted the presence of well-defined functional categories. Those most represented included ECM, Tropomyosins, Cytoskeleton, Carbohydrate metabolism, and Proteins folding. However, although less represented, other interesting proteins involved in Proteolysis regulation, Redox homeostasis, Histone proteins, and Annexins were found. All these categories agreed with those (Biological Process, Molecular Function, and Cellular Component) most enriched by considering the global characterizes protein profile (Figure S1); interestingly, a relevant enrichment of proteins involved in chaperone-mediated protein folding emerged from this analysis. A major level of detail concerning functional categories and proteins most relevant in the Lyosecretome from equine MSCs was reached by reconstructing and processing, at a functional and topological level, an *Equus caballus* protein–protein interaction (PPI) network; it was reconstructed exploiting the high sequence homology (>87%) between *Equus caballus* and *Homo sapiens* protein sequences (Figure S2).

**Figure 9:**
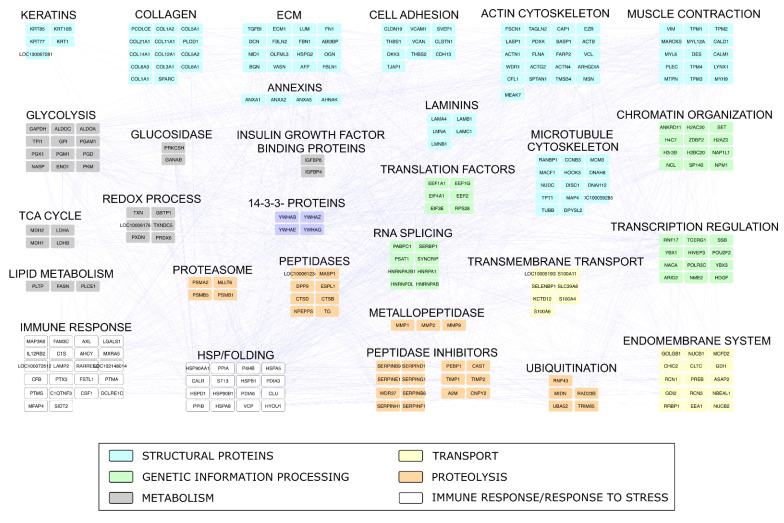
Equus caballus PPI network reconstructed by homology with Homo sapiens. The network was built by STRING Cytoscape APP starting from proteins secreted by mesenchymal stem/stromal cells and identified at least two out of seven replicate analyses.

**Figure 10:**
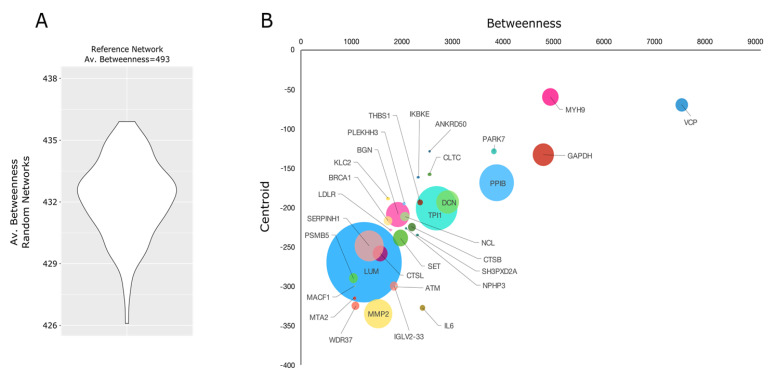
PPI network hubs in PPI network reconstructed from proteins secreted by *Equus caballus* mesenchymal stem/stromal. (**A**) Violin plot reporting the average Betweenness in Random networks. (**B**) PPI network hubs were selected considering Betweenness, Centroid, and Bridging (proportional to circle size) centralities; proteins were defined as hubs if all centrality values were above the average values calculated on the whole network.

The Equus caballus PPI network reconstructed starting from proteins secreted by mesenchymal stem cells counted 269 nodes and 2016 edges. They were clustered in 29 functional/topological modules (Figure 9) further classified in 6 macro-areas, including Structural proteins, Genetic Information Processing, Metabolism, Transport, Proteolysis, and Immune response/Response to stress. Following the Biological processes, Molecular Functions, and Protein families that emerged from network analysis, as well as the Lyosecretome therapeutic properties observed, we focused our attention on proteins involved in inflammatory processes and response to a bacterium (Table S2); specifically, six proteins (IL10RA, LTA4H, MXRA5, RARRES2, TEK, ANXA1) were annotated by “Anti-inflammatory activity”, six proteins (C1QTNF3, ENPP3, GAPDH, KRT1, SERPINF1, SYNCRIP) by “Negative regulation of inflammation”, while 35 were more generically “Involved in inflammatory response”. In addition, seven proteins (RARRES2, NOD1, SERPINE1, H2BC20, SERPINB9, PENK, EPX) were annotated to be involved in “Response to bacterium”. Of note, SERPINF1 and ANXA1 were also found among the most abundant proteins, suggesting a putative active role as a candidate in exerting anti-inflammatory properties (Figure 8C). With the purpose to shed light on other proteins with a potential regulatory role and functionally capable of holding together communicating proteins by signaling mechanisms, we selected a group of proteins that occupy specific network positions and that are defined as hubs (Figure 10); within this group, we found proteins “Involved in inflammatory reaction”, such as IKBKE, ADCY1, MMP2, PARK7, THBS1, and IL6, which is also annotated to be involved in the defense response against the bacterium.

## 3. Discussion

This study aimed to propose a procedure for a scalable production of equine Lyosecretome, define its characteristics, and pave the way for its use in in vivo preclinical studies in animals affected by musculoskeletal disorders. To this aim, secretome production was induced by serum starvation of expanded AD-MSCs, and then the freeze-dried secretome was characterized in terms of qualitative and quantitative parameters. Experimental evidence indicates that MSCs can be isolated from almost every tissue of the body [15], even if it is still unclear whether cells derived from different sources share the same biological and therapeutical features. However, compared to the Lyosecretome prepared from MSCs isolated from human adipose tissue, we found similar categories, including genetic information processing, immune response, and stress response. Adipose tissue and bone marrow are the preferred sources to isolate and expand MSCs for equine clinical applications [13]. In our study, AD-MSCs were used for the preparation of Lyosecretome.

Ultrafiltration was chosen for the easy scalability to prepare large batches because it presents several advantages compared to other techniques commonly used to isolate MSC-secretome, including the affordable costs and the shorter time requested by the entire process [16]. Furthermore, this technique grants the choice between filtration modules with different molecular weight cut-offs (MWCO), allowing the preferred retention based on the desired yield in small vesicles (as, for example, extracellular vesicles) or the whole secretome. Other conventional methods commonly used for isolating EVs, such as ultracentrifugation, present several limitations regarding the possible damage of EVs membranes, due to the high g-force required by the centrifugation steps [17]. In the current study, we selected a relatively low MWCO able to retain the whole secretome to ensure a large-spectrum therapeutic potential to the preparation, even though several studies support the hypothesis that the EVs fraction alone might be sufficient to promote healing of the injured tissues [18]. A biotechnological product such as secretome can be thermolabile, and thus it cannot be dried applying thermal methods, responsible for possible modification and/or degradation of bioactive components (e.g., protein). For this reason, to reach a water-free product, the lyophilization process was considered. Lyophilization guarantees the maintenance of sterile conditions when changing from liquid to a powder state; moreover, the final product is easy to store and requires a short reconstitution time of the dried product; long-term stability is also provided [19]. Unfortunately, this technique has several limitations: Costs, limited capacity, long processing time (it lasts approximately 4 days), and difficulties in the “scale up” from laboratory to industrial production. Thus, its use is justified when the product’s value is sufficiently high, such as producing biotechnological drugs, vaccines, vitamins, antibiotics, liposomes, and oncological products. The freeze-drying method creates freezing and drying stresses that can alter the stability of the biological product; hence, before proceeding, the addiction of a cryoprotectant is needed. The cryoprotectant guarantees the protection of both protein and vesicles lipidic layer from the damages of ice crystals formed during the freezing step and inhibits vesicle size alteration by aggregation. In this regard, mannitol is the most suitable cryoprotectant due to its important stabilizing effects on freeze-dried pharmaceutical products [19]. Mannitol properties have been widely discussed and investigated [20,21]. Its role is to ensure long-term stability, easy reconstitution, and maintenance of biological features and activities. However, the use of mannitol still remain controversial and many other excipients, such as trehalose, can be adopted instead [22]. In the current work, mannitol was able to avoid vesicles/particles aggregation as revealed by NTA analysis; moreover, it prevented vesicle structure modification in terms of morphology as shown by SEM analysis. This latter aspect was also ensured by applying ultrafiltration process for the secretome isolation, rather than ultracentrifugation, which can possibly break-up vesicles membrane due to the high shear forces.

FTIR spectra revealed similar bands for each of the three batches, where the presence of lipids and protein content was confirmed. Overall, all samples’ spectra were overlapping, thus ensuring the reproducibility of the batch production process. DSC has been employed to demonstrate the purity, polymorphic form, melting point, and thermal behavior of EV lipid bilayers, which can be affected in terms of stability under adverse conditions [23]. The lyophilization process was conducted successfully for all batches, as confirmed by TGA analysis, with a very low water residual and good thermal stability.

Regarding the analysis performed by the ExoView, the method is based on a direct-biomarker association that captures particles into a chip. The technique allows discriminating whether a protein is expressed on the surface of the exosome or in the exosome by fluorescence analysis of the captured particles. Since a specific chip for analysis of equine secretome is not available, the microarray specific to human proteins was used. Although not validated for equine species, the chip is based on highly conserved proteins, and a cross-reaction is plausible. We observed differences between the analysis of the three batches. The number of exosomes presenting a double correlation anti-CD-81/CD-9 human antibody was higher than the other correlations; moreover, CD-9 binding is probably the most affine to horse secretome’s surface protein due to a higher protein match.

The anti-elastase activity tested on equine Lyosecretome provides the rationale for using secretome in regenerative medicine [24]. High protease expression is a feature commonly found in several musculoskeletal disorders contributing to their onset and evolution. In this context, protease inhibitors work, contributing to restoring extracellular matrix homeostasis. Our results showed a dose-dependent trend for each batch. Significant variability was observed between the different preparation, probably caused by an intrinsic biological variability between the cell populations used for their preparation.

In vitro metabolic activity assays are the gold standard for studying cell viability and proliferation. In the present work, MTT assay was performed on four different cell types, i.e., AD-MSCs, SF-MSCs, tenocytes, and chondrocytes, considered potential targets for the use of Lyosecretome in musculoskeletal disorders. No cytotoxic effect was observed after Lyosecretome treatment in any of the four different cell lines, independently of the used concentration. Lyosecretome was able to maintain cell replication when compared to serum-free medium. A dose-dependent effect was observed for each cell line, and statistically significant differences were observed as a function of the cell type. Cell metabolic activity ranged from 30% to 75% compared to medium supplemented with FBS, with the higher stimulus observed at a Lyosecretome concentration of 200 mg/mL. Interestingly, at this concentration, tenocytes, chondrocyte, and SF-MSCs were more responsive than AD-MSCs, reaching a plateau at 200 mg/mL.

The effect observed following Lyosecretome treatment, in particular elastase inhibition, could be attributable, at least partially, to its protein content. In fact, in addition to proteins involved in stress response, including the Heat Shock Proteins, we found protease/peptidase inhibitors among the protein classes better represented. In particular, the putative relevant role of some of them, such as SERPINs, was also suggested by their presence among the most abundant proteins (SERPIN1F) and proteins defined as hubs (SERPINH1). Moreover, about 10% of the identified proteins were annotated to be involved in the inflammatory response. In addition to SERPINE1, annotated to also be involved in “Response to bacterium”, the presence of proteins with anti-inflammatory activity (IL10RA, LTA4H, MXRA5 [25], RARRES2 [26,27], TEK, ANXA1 [28]) emerged. Some of them, including MXRA5 (Chemerin), have been previously associated with osteoarthritis, and anti-inflammatory and anti-fibrotic properties have been proposed for this protein suggesting its potential role in chronic inflammation [29]. In this scenario, MACF1 has also been detected in Lyosecretome; this protein plays a core function in wound healing cell migration [30,31], primarily due to its ability to coordinate interaction between actin, microtubule, and cell junctions. Concerning the cytokine profile, several proteins with cytokine/chemokine activity have been observed in the Lyosecretome, i.e., IL-6, IL-7, CSF-1, IL10RA, IL12RB2, CXCL6, GDF7, and Wnt5B. IL-6 and IL-7 are two pro-inflammatory cytokines involved in MSCs immunomodulation [32,33]. The presence of IL-6 agrees with a recent paper by Bundgaard et al. that also demonstrated the presence of CXCL6 in equine bone marrow-derived MSCs [34]. Pro-inflammatory and chondrogenic treatments modulated the expression of both proteins. IL6, acting as a pro-inflammatory mediator, contributes to host defense during infection and tissue injury, but anti-inflammatory properties have also been suggested depending on the presence of other cytokines [35]. CSF-1 plays a role in inflammatory processes and promotes bone regeneration and mobilization of vascular and osteogenic progenitor cells [36]. CSF-1 presence has also been demonstrated in equine bone-marrow-derived MSCs secretome [34]. GDF7 and WNT5B are two secreted signaling proteins whose role has been suggested in tenogenic (GDF7) and osteogenic and chondrogenic (WNT5B) differentiation pathways of MSCs [37,38]. IL10RA mediates the immunosuppressive signal of interleukin 10, an anti-inflammatory cytokine, already observed in bone marrow-derived human MSCs secretome [39].

Highly expressed proteins belonging to the class I small leucine-rich proteoglycan (SLRP) family, including LUM (lumican), DCN (decorin), and BGN (biglycan), were found among proteins defined as hubs. SLRP family comprises a group of extracellular matrix components distributed in most tissue whose gene expression is modified in OA [40]. Lumican, one of the most abundant proteins found in the secretome, regulates collagen fibril organization, and its expression is reduced in OA cartilage [40]. Interestingly, lumican is a major component of the corneal stroma, and its therapeutic use has been proposed for ocular lesions [41]. Furthermore, lumican is considered a key effector in normal wound repair [42]. Decorin plays a role in fibril assembly in tension-bearing tissues. It may contribute to stabilizing the cartilage matrix in OA [43]. Decorin upregulation has been proposed as an attempt to reduce aggrecan fragmentation in post-traumatic OA, and decorin-based therapeutics have been suggested to attenuate OA progression [44]. Concerning biglycan, this extracellular matrix component contributes to the regulation of chondrogenesis and ECM turnover in OA [45]. Interestingly, the Lyosecretome prepared from equine adipose tissue-derived MSCs shares with the equine bone-marrow-derived MSCs secretome described by Bundgaard et al. [34] a number of proteins related to cartilage biology, i.e., LUM, DCN, COMP (cartilage oligomeric matrix protein), SCRG1 (stimulator of chondrogenesis-1), EPYC (Epiphycan), GAS6 (growth arrest specific 6).

Regarding Lyosecretome as a pharmaceutical product, the following acceptance criteria were defined: (i) Fulfilment of specific requirements by the cellular source: Viability over 98%, capable for trilineage differentiation and negative for bacterial/viral contamination. (ii) Protein and lipid content were between 10–13 and 0.6–1 μg per mg of powder, respectively, mean EV size in the range of 100–200 nm, EV concentration in the range of 1–4 × 10^8^ particles when re-suspended in dH_2_O at 1 mg/mL, expression of CD-9, CD-63, and CD-81 markers, negative for bacterial/viral contamination, water content less than 2%, fulfilment of the criteria required for freeze-dried (cake aspect) and injectable formulations (osmolarity, pH, absence of visible particles after resuspension in physiological solution). The assessment on the final product has to be performed by characterization techniques that can be validated at pharmaceutical grade and have an acceptable cost to be routinely implemented [14,46,47]. (iii) Functional aspects should be included to demonstrate that the final product, after having undergone all the isolation, purification, and formulation processes, maintains biological activity. In detail, anti-elastase activity was higher than 20% at 20 mg/mL in dH_2_O. Finally, due to the limited experience in preparing equine Lyosecretome (these are the first scaled-up batches prepared following ISO 9001:2018 for validation) the source of variability cannot be fully investigated yet. With a more consistent number of batches prepared, it will be possible to adopt specific measures to minimize the batch-to-batch variability.

## 4. Materials and Methods

The Lyosecretome production was made at an accredited facility to produce and control veterinary drugs for clinical use (Istituto Zooprofilattico Sperimentale Lombardia and Emilia-Romagna (IZSLER, Brescia, Italy). The production protocol has been approved by the Italian Ministry of Health (Prot. n. 0000778 del 15/01/2020 7.1.2.0.0.0/17/2019—AGD 809). Culture media, trypsin, and antibiotics used for cell cultures were purchased from Euroclone (Milan, Italy). A commercial platelet lysate kit (PL) was obtained from Carlo Erba Reagents (Milan, Italy). Fetal Bovine Serum (FBS) was purchased by Gibco-Thermofisher (Milan, Italy). Chemicals such as mannitol, bovine serum albumin, Nile Red, acetone, collagenase, and phosphatidylcholine were obtained from Merck Life Science S.r.l. (Milan, Italy) unless mentioned.

### 4.1. Lyosecretome Production

#### 4.1.1. Mesenchymal Stem/Stromal Cell Isolation

Allogeneic AD-MSCs from equine species were obtained from the biobank at Istituto Zooprofilattico Sperimentale Lombardia and Emilia-Romagna (IZSLER) (Brescia, Italy). The clinical data related to the cells stored in the biobank as gender, age, the weight of the donor, and cell passage number, were recorded by the IZSLER. In detail, the secretome was produced by three donors. The donors were 10 years old, male, and the breed is unknown. The samples were tested for Equine Herpesvirus, Equine Arterivirus, Flavivirus, and Lentivirus (antibodies screening). All the samples were collected from animals killed at the slaughterhouse following the related Italian Law. Adipose tissue digestion was performed at 37 °C, 5% CO_2_ using type II collagenase 0.075% (*w/v*) solubilized in Phosphate Buffer Saline (PBS) with Ca^2+^ and Mg^2+^ supplemented with penicillin (50 U/mL), streptomycin (20 µg/mL), and amphotericin B (2.5 µg/mL). After 1 h, Dulbecco’s modified Eagle’s Medium (DMEM) with 10% (*v/v*) of equine platelet lysate was added to the cell suspension in a 1:1 ratio. The digested tissue was filtered with 70 μm filters and centrifuged at 600× *g* for 5 min. The recovered stromal vascular fraction was grown in a monolayer (100,000 cells/cm^2^) in DMEM, enriched with 10% (*v/v*) platelet lysate, penicillin, streptomycin, and amphotericin B. On reaching sub-confluence, the MSCs were detached from the flask with 0.05% (*w/v*) trypsin-EDTA, counted, seeded in flasks (10,000 cells/cm^2^) at 37 °C and 5% CO_2_, and amplified. Upon reaching sub-confluence, the cells were ready for the induction of secretome production. All cells used were at passage 1 and tested for trilineage differentiation and microbiological controls.

#### 4.1.2. Mesenchymal Stem/Stromal Cell-Derived Secretome Production

Secretome production was induced by platelet lysate starvation of MSCs. In detail, the culture medium was removed and the cells were repeatedly washed (at least three times) with PBS without Ca^2+^ and Mg^2+^ to eliminate any residual platelet lysate [48]. MSCs were then cultured in a DMEM medium without platelet lysate for 24 h, replacing the culture medium after the first 9 h. Both conditioned media (collected at 9 and 24 h, respectively) were blended; MSCs were detached with trypsin-EDTA and counted in a Burker chamber.

#### 4.1.3. Ultrafiltration

The collected conditioned media was firstly centrifuged at 3500× *g* for 10 min to eliminate cell fragments and apoptotic bodies. Subsequently, supernatants were collected, and the ultrafiltration process was performed applying a tangential flow filtration using KrosFlo^®^ Research 2i system (Spectrum Laboratories, Milan, Italy), using Molecular Weight Cut Off (MWCO) of a 5 kDa filtration module (Spectrum Laboratories, Milan, Italy). For the ultrafiltration process, all the instrument components were sterilized to operate in aseptic conditions under a laminar flow hood. The ultrafiltration process consists of two phases, at first allowing to concentrate the sample, and then to diafilter with sterilized and ultrapure water; according to manufacturer’s guidelines, during each step, the shear rate of the feed stream was kept between 2000 s^−1^ and 6000 s^−1^, while the trans-membrane pressure index was kept at maximum 5 psi. The concentration step led to a concentration of 0.5 × 10^6^ cell equivalents per mL. The evaluation of the process scale-up was calculated as average liters per m^2^ per h:

L/m*^2^*/h = permeate flux (mL/min)/cartridge superficial area (m*^2^*) 0.06

#### 4.1.4. Secretome Freeze-Drying

As the elective cryoprotectant, mannitol was chosen for this study, and it was mixed with the purified secretome at the concentration of 0.5% (*w/v*). The resulting solution was at first frozen at −80 °C, and then a conservative freeze-drying process was adopted (Heto PowerDry PL3000) at 8 × 10^−1^ mbar and −50 °C for 72 h. The lyophilized secretome was kept at −20 °C up to the time of use (maximum 9 months). Before and after the freeze-drying process, Lyosecretome yield (mg) was established, and cell equivalents per mg were calculated by dividing the total cell number used for production and the gained milligrams of Lyosecretome.

### 4.2. Lyosecretome Characterization

#### 4.2.1. Residual Humidity and Osmolarity

After the samples rebalance at room temperature, residual humidity was determined by the Coulometric Titrator HI904 (Hanna Instruments). The titration was conducted two times on each vial (*n* = 3 vials). After reconstitution of 10 mg of lyophilized product in 2 mL saline (0.9% *w/v* NaCl in water) at 37 °C, the osmolarity was measured using a micro osmometer (Precision System Inc., Natick, MA, USA). The pH of the reconstituted product was measured by pH meter (Mettler-Toledo, Columbus, OH, USA).

#### 4.2.2. Fourier Transform Infrared Spectroscopy (FT-IR)

FT-IR spectra of Lyosecretome were obtained using a Spectrum One Perkin-Elmer spectrophotometer (Perkin Elmer, Wellesley, MA, USA) equipped with a MIRacle™ ATR device (Pike Technologies, Madison, WI, USA). The IR spectra in transmittance mode were collected in the spectral region of 650–4000 cm^−1^ due to the accumulation of 64 scans with a resolution of 4 cm^−1^.

#### 4.2.3. Thermal Characterization

Differential scanning calorimetry (DSC) analysis measured temperature and enthalpy values with a Mettler STAR^e^ system (Mettler Toledo, Columbus, OH, USA) equipped with a DSC81^e^ Module and an Intracooler device (Jukabo FT 900) for sub-ambient temperature analysis. Firstly, the instrument was previously calibrated with Indium as a standard reference. Outcome curves were achieved recording on about 3 mg of samples in 40 μL sealed aluminum pans with pierced lids (method: −30–350 °C temperature range; heating rate 10 K min^−1^; nitrogen air atmosphere flux 50 mL min^−1^). Experiments were performed in triplicate.

Thermogravimetric analysis (TGA) was measured with a Mettler STAR^e^ system equipped with a TGA/DSC1. Firstly, the instrument was calibrated with Indium as a standard reference. Outcome curves of the mass losses were obtained recording on 3–4 mg of samples in 70 μL alumina pans (method: 30–350 °C temperature range; heating rate 10 K min^−1^; nitrogen air atmosphere flux 50 mL min^−1^). Experiments were performed in triplicate.

#### 4.2.4. Morphology Investigation by Scanning Electron Microscopy (SEM)

Lyosecretome morphology has been investigated by SEM with a Zeiss EVO MA 10 (Carl Zeiss, Oberkochen, Germany). For the analysis, the samples were coated with a gold-sputter under argon.

#### 4.2.5. Particle Size Distribution

The particle size distribution of Lyosecretome was evaluated by performing Nanoparticle Tracking Analysis (NTA) using NanoSight NS 300 (Malvern Instruments, Malvern, UK). In detail, the freeze-dried powder was resuspended in 1 mL of deionized water (1 mg/mL) and analyzed with a dilution factor of 1:10. All measurements were repeated for 6 cycles of 60 s each, using NanoSight protocols.

#### 4.2.6. Microvesicles Surface Biomarkers by ExoView Analysis

ExoView (Alfatest, I) is an innovative test to determine the size and number of exosomes in a sample by biomarker detection. The Tetraspanin Human standard Kit with a thin layer of CD63, CD81, and CD9 was chosen, and solutions for sample dilution were supplied by the Nano View Bioscience company. The analysis method could be divided into two macro steps: Interferometric imaging that gives information about the exosome’s number and exosome’s size; and fluorescence analysis that offers the intensity of a positive-marker protein. Samples were prepared in triplicate and diluted according to the protocol suggested by the supplier for the tetraspanin kit (NanoView Biosciences; EV-TETRA) and incubated for 16 h on ExoView tetraspanin chips. The chips were coated with a fluorescent antibodies mixture of anti CD81, CD63, or CD9, placed on an orbital shaker at 500 rpm for 1 h, and then washed four times. Chips were transferred into a Petri plate with MilliQ water for the final washing step and, after drying with an adsorbent paper, placed onto ExoView instrument’s chip holder and analyzed. The results are the average of three determinations.

#### 4.2.7. Total Protein Quantification

At the end of the lyophilization process, Lyosecretome protein quantification was determined by using the BCA-Protein Assay Kit (Thermo Fischer Scientific, Milan, Italy) following the manufacturer’s specifications. The absorbance–concentration calibration curve was generated using bovine serum albumin (BSA) standards. Working reagent solution was added to each sample and standard (ratio 1:1) and then incubated at 37 °C for 2 h before measuring the absorbance at 562 nm with a microplate reader (Synergy HT, Milan, Italy). The Lyosecretome protein amount was estimated as a plot function comparing concentration vs. absorbance obtained for the standard protein solutions, using a third-order polynomial equation, with *R*^2^ = 0.99. Results are reported as µg of protein per mg of Lyosecretome. Each sample was tested in triplicate.

#### 4.2.8. Total Lipid Quantification

To prepare the Nile Red stock solution, the Nile Red powder was solubilized in acetone (3.14 M), avoiding light exposure, and kept at 4 °C. The stock solution was diluted 100× in PBS (pH = 7.4) before use, and 10 µL of it were incubated with 90 µL of samples. After 5 min, the relative fluorescence was calculated by Sinergy HT (530/25 excitation and 645/40 emission). The Lyosecretome lipid amount was estimated as a plot function comparing concentration vs. absorbance obtained for standard phosphatidylcholine solutions, using a third-degree polynomial equation, with *R*^2^ = 0.99. Results are reported as µg of lipid per mg of Lyosecretome. Each sample was tested in triplicate.

#### 4.2.9. Sterility Test and Microbiological Control

Each batch was investigated for sterility, endotoxins, Mycoplasma, and microbiological contaminations, as described in the current version of European Pharmacopoeia. Briefly, sterility and microbial examinations were performed as indicated in EuPh 2.6.27 and 2.6.1 chapters, respectively. Furthermore, a bacterial endotoxins evaluation was carried out by the Limulus Amebocyte Lysate test (EuPh 2.6.14), a chromogenic kinetic method, and measured as an Endotoxin Unit (EU). The test was performed following the manufacturer’s instructions. Finally, mycoplasma contamination was investigated by performing specific tests (NAT test, EuPh 2.6.7.).

### 4.3. In Vitro Potency Test

#### 4.3.1. Elastase Inhibitory Assay

The in vitro inhibitory effect of equine Lyosecretome on the elastase enzyme was evaluated using the method previously described in the literature, with some modifications [49]. Pancreatic porcine elastase (Merck Life Science S.r.l.) was solubilized in phosphate buffer pH 6.8 (0.5 IU mL^−1^). The substrate N-succinyl-Ala-Ala-Ala-p-nitroanilide (Merck Life Science S.r.l.) was dissolved in TRIS buffer until a final concentration of 0.41 mmol L^−1^ was reached. All the tested concentrations (2, 5, 10, 20 mg mL^−1^) were incubated with the enzyme for 20 min, and, consequently, the substrate was added right before reading the microplates to begin the reaction. The kinetic reaction was monitored by spectrophotometric analysis (Synergy HT) at the absorbance of 410 nm for 35 min (measurements were made every minute). The reaction mixture in the absence of sample was used as a negative control, while the epigallocatechin gallate (EGCG) (Merck Life Science S.r.l.) was used as a positive control. The absorbance value of each sample was subtracted from the absorbance of the blank mixture (sample without the enzyme and substrate). Analyses were performed in triplicate. The inhibition rate was reported as a percentage of anti-elastase activity and calculated as follows:

Activity (%) = [(A_CTR_ − A_samp_)/A_CTR_] × 100

where A_CTR_ is the negative control absorbance and A_samp_ is the Lyosecretome sample absorbance.

#### 4.3.2. Proliferative Test

The proliferative effects of Lyosecretome were evaluated on four different equine cell lines: Chondrocytes, tenocytes, synovial fluid, and adipose tissue-derived MSCs. Tissue samples were collected at the local abattoir following the related Italian Law from three healthy 5- to 10-year-old male horses of unknown breed, ensuring the absence of viral and bacterial pathologies. AD-MSCs were isolated and expanded as described above (see paragraph 2.1.2).

##### Synovial Fluid Cells Collection and Expansion

The synovial fluid was collected from the metacarpophalangeal (fetlock) joint. Following trimming and disinfection, 4–5 mL of fluid were collected with a sterile syringe, transferred in a 50 mL falcon-type tube, and diluted 1:10 with sterile phosphate-buffered saline (PBS). After accurate mixing, the tube was centrifuged at 500× *g* for 20 min. The cell pellet was resuspended in 2 mL of DMEM supplemented with penicillin (50 U/mL), streptomycin (25 µg/mL), and amphotericin B (2 µg/mL) and transferred in two 25 cm^2^ culture flasks. After 48 h, the medium was changed. The attached cells were expanded until they reached about 80% confluence when they were trypsinized and expanded at a 1:3 ratio. Cells were used for further experiments at passage P3–P4.

##### Tenocytes Isolation and Expansion

Tissue samples of superficial digital flexor tendon (SDFT) were collected in sterility and dissected in 2–3 mm^3^ pieces with a scalpel blade, removing tendon sheath. The tissue was digested overnight in 1 mg/mL collagenase II (5 mL/g of tissue) at 37 °C. After digestion, the cell suspension was strained (40 µm nylon mesh) and then centrifuged at 900× *g* for 15 min. The cell pellet was resuspended in 2 mL of DMEM supplemented with 1% (*v/v*) penicillin (100 U/mL), streptomycin (100 µg/mL), and amphotericin B (2 µg/mL). Cell viability was assessed by Trypan blue staining. Cells were then transferred in 25 cm^2^ culture flasks (10,000 cm^2^) and expanded in DMEM at 37 °C, 5% CO_2_. Upon reaching P3–P4, cells were used for further experiments.

##### Chondrocytes Isolation and Expansion

Cartilage tissue samples were collected from the metacarpophalangeal joint. The joint was exposed in sterility, and about 1 g of cartilage was collected with a scalpel blade in small pieces. Tissue samples were digested overnight in 5 mL of collagenase type II (0.1%, *v/v* in DMEM) at 37 °C [2]. After digestion, the cell suspension was strained (40 µm nylon mesh) and centrifuged at 180× *g* for 15 min. The cell pellet was resuspended in DMEM supplemented with 1% (*v/v*) penicillin (100 U/mL), streptomycin (100 µg/mL), and amphotericin B (2 µg/mL) and then seeded with the same medium in 25 cm^2^ culture flasks (10,000 cells/cm^2^). Cells were used for in vitro metabolic activity assay at passage P3–P4.

##### Proliferative Test by MTT

The MTT assay was performed to assess cell metabolic activity in the four different cell lines reported above (AD-MSCs, SF-MSC, tenocytes, and chondrocytes) as potential targets for the use of Lyosecretome in osteoarticular diseases. In detail, 10,000 cells/well were plated in 96-well plates in DMEM supplemented with 10% Fetal Bovine Serum (FBS); three replicates were prepared for each treatment. After 24 h, the medium was replaced with serum-free DMEM containing Lyosecretome at the concentrations of 400,000, 200,000, and 100,000 cells/well. Cells were cultured for a further 48 h, and then the MTT test was performed as previously reported [50]. Briefly, the cells’ optical density (OD) after MTT treatment was measured at 570 nm and 670 nm (reference wavelength). The cell metabolic activity percentage was calculated as 100 × (ODsample/ODcontrol). Cells cultured with DMEM containing 10% FBS were considered a positive control (ODcontrol), while the cells cultured without serum (serum-free medium) were considered a negative control. The MTT assay was repeated with four different cell preparations for each cell line.

### 4.4. Proteomic Investigation

#### 4.4.1. LC-MS/MS Analysis

Two milliliters of sample were concentrated to 100 µL in a vacuum concentrator at 60 °C, and the protein concentration was assessed using the SPN^TM^–Protein Assay kit (G-Biosciences, St. Louis, MO, USA); 50 µg of proteins were added with 0.2% of Rapigest and held at 100 °C for 20 min. The sample was digested overnight at 37 °C by adding sequencing-grade modified trypsin (Promega, Madison, WI, USA) at an enzyme/substrate ratio of 1:50 (*w*/*w*). An additional aliquot of trypsin (enzyme/substrate ratio of 1:100 *w/w*) was added in the morning (4 h, 37 °C). The addition of 0.5% trifluoroacetic acid stopped the enzymatic reaction. Digested proteins were desalted using PepClean C-18 spin columns (Pierce Biotechnology, Inc., Rockford, IL, USA), concentrated, and finally suspended in 20 µL of 0.1% (*v*/*v*) formic acid.

The sample was analyzed using two LC-MS/MS platforms for a total of seven technical replicates. The first LC-MS/MS system (*n* = 3) was equipped with an Eksigent nanoLC-Ultra 2D System (Eksigent, part of AB SCIEX, Dublin, CA, USA) coupled with a hybrid ion trap-Orbitrap mass spectrometer (LTQ Orbitrap XLTM ETD; Thermo Fisher Scientific, Inc., Milan, Italy). The loading pump runs in isocratic mode with 0.1% formic acid in water for 10 min at a flow rate of 3 µL/min; the gradient pump runs a 125-min gradient of 5 to 95% of eluent B (5–40% B in 110 min, 40–95% B in 15 min; eluent A, 0.1% formic acid in water; eluent B, 0.1% formic acid in acetonitrile) at a flow rate of 300 nL/min through the column (75 µm × 15 cm ChromXP C18-CL 3 µm, 120 Å). Eluting peptides were electrosprayed directly into a hybrid ion trap-Orbitrap mass spectrometer (LTQ Orbitrap XLTM ETD; Thermo Fisher Scientific, Inc., Milan, Italy), equipped with a nanospray ion source. The spray capillary voltage was set at 1.7 kV, and the ion transfer capillary temperature was maintained at 220 °C. Full mass spectra were recorded in the positive ion mode over a 400–1600 *m*/*z* range, with a resolving power of 60,000 (full width at half-maximum). This step was followed by five low-resolution MS/MS events that were sequentially generated in a data-dependent manner on the top five ions selected from the full MS spectrum, using dynamic exclusion for the MS/MS analysis. In particular, the MS/MS scans were acquired by setting normalized collision energy of 35% on the precursor ion and, when a peptide ion was analyzed twice, applying an exclusion duration of 0.5 min.

The second LC-MS/MS system (*n* = 4) was equipped with a two-dimensional micro-high-performance liquid chromatography system (Surveyor HPLC; Thermo Fisher Scientific, Inc., San Jose, CA, USA) coupled online to an LTQ mass spectrometer (Thermo Fisher Scientific, San Jose, CA, USA). Ten microliters of peptide mixtures were concentrated and desalted online by C18 traps loaded on a 10-port valve before final separation on the capillary reversed-phase column (Biobasic-C18, 0.180 i.d. ×100 mm, 5 μm particle size, Thermo Fisher Scientific, San Jose, CA, USA). The flow rate was 100 μL/min, which was split to achieve a final flux of 2 μL/min. Peptides were separated with the following eluents: (A) 0.1% formic acid in water; (B) 0.1% formic acid in acetonitrile; the gradient profile was 5–40% B in 110 min, 40–95% B in 15 min; the flow rate on C-18 column was 1 μL/min. The peptides eluted from the C18 column were directly analyzed with an LTQ mass spectrometer (Thermo Fisher Scientific, San Jose, CA, USA) equipped with a nano-ESI source. Full MS spectra were acquired in positive mode over a 400−2000 *m*/*z* range, followed by five MS/MS events sequentially generated in a data-dependent manner on the first five most intense ions selected from the full MS spectrum (collision energy 35%) and using dynamic exclusion for MS/MS analysis.

#### 4.4.2. Proteomic Data Processing

The experimental MS/MS spectra produced by LTQ and LTQ Orbitrap XLTM ETD mass spectrometers were matched against the in silico tryptic peptide sequences of the Equus caballus protein database retrieved from UNIPROT in March 2021 (44484 protein sequences). Data processing was performed by Discoverer 2.5 software, based on the SEQUEST HT algorithm [51]. Peptide and protein assignment was made according to specific guidelines [52]. The following criteria were used for peptide identification: Parent mass tolerance of 50 ppm and 200 ppm was set for LTQ Orbitrap XLTM ETD and LTQ, respectively; while for both peptides, fragment mass tolerance was set to 0.8 Da, respectively. Missed cleavage sites per peptide were set to 3. The percolator node was used with a target-decoy strategy to give a final false discovery rate (FDR) ≤ 0.01 (strict) based on q-values, considering a maximum ΔCN of 0.05. Only peptides with a minimum peptide length of 5 amino acids, confidence at “Medium” level, and rank 1 were considered. Protein grouping and strict parsimony principles were applied. To evaluate the correlation among replicate analyses, Spearman’s rank correlation was computed by JMP15.2 SAS software. To provide a rank list of the most abundant proteins in the analyzed samples, the average Spectral count (SpC) of each protein was normalized on MW as previously reported [53]. Functional Annotation Tool of DAVID database [54] was used to characterize the most enriched molecular function (MF), biological process (BP), and cellular component categories; specifically, background = Equus caballus, count > 5 and *p* < 0.01 were set.

An *Equus caballus* protein–protein interaction (PPI) network was built by homology with *Homo sapiens,* as previously reported [55]. Proteins identified in at least 2 out of 7 replicate analysis were combined with the *Homo sapiens* PPI network retrieved from the STRING database [56]. Only experimentally and database-defined PPIs with a score > 0.15 and 0.35, respectively, were considered. The resulting sub-network was visualized and analyzed by Cytoscape and its plugins [57]. It was processed at the functional level using STRING Cytoscape APP [56]. Network topological analysis was performed by Centiscape2.2 Cytoscape plugin as previously reported [58]; betweenness, centroid, and bridging centralities were evaluated. A set of nodes with values above the average calculated on the whole network were defined as hubs [59]. The statistical significance of topological results was tested by considering randomized network models; they were reconstructed and analyzed by an in-house R script based on VertexSort (to build random models), igraph (to compute centralities), and ggplot2 (to plot results) libraries.

### 4.5. Statistical Analysis

Raw data were processed through STATGRAPHICS XVII (Statpoint Techonologies, Inc., Warrenton, VA, USA). A general linear analysis of variance model (ANOVA) was generated to evaluate the data. The function was then followed by an LSD test to estimate the differences between means. Each batch was processed, considering protein and lipid content as the response variable and the batch number as the fixed factor. To evaluate cell proliferation, the cell metabolic activity was set as the response variable and the Lyosecretome concentration as a fixed factor. Statistical significance was set at *p* < 0.05.

## 5. Conclusions

This study provides the proof-of-principle of the feasibility of a clinical-grade injectable freeze-dried formulation containing the secretome from equine adipose tissue-derived mesenchymal stem/stromal cells. The technological production process has proven to be consistent, and a panel of tests for quality control and batch release has been defined. Lyosecretome exerts a direct activity on different musculoskeletal cell types such as tenocytes, chondrocytes, and tissue-resident MSCs; also, proteomic composition suggests a possible role of Lyosecretome for regenerative medicine applications. In detail, our findings suggest the therapeutic role in musculoskeletal diseases and wound healing of many proteins found in the equine secretome, including SLRP family members. Certainly, a more in-depth evaluation of the role of these proteins could be performed, and it will be the object of future investigations. Although extensive in vitro characterization is needed, this work paves the way for the clinical use of Lyosecretome: The proof-of-concept for manufacturing clinical-grade equine Lyosecretome has been provided, and prototypes are now available to evaluate its safety and efficacy in the treatment of horse musculoskeletal diseases.

## Figures and Tables

**Table 1:** Lyosecretome batch production from three different equine AD-MSCs lines.

Batch n.	Total Cell Number × 10^6^	Cell Viability (%)
1	224	99
2	170	98
3	65	99

**Table 2:** Lyosecretome microbiological test: Sterility, bacterial endotoxin, and Mycoplasma.

Batch n.	Sterility	Bacterial Endotoxin	Mycoplasma
1	compliant	3.4 Eu/mL	no presence
2	compliant	3.3 Eu/mL	no presence
3	compliant	3.4 Eu/mL	no presence

**Table 3:** Particle size distribution and concentration of batch 1 and 2 (mean values ± standard deviation, *n* = 3).

Batch n.	Mean (nm)	Mode (nm)	d_10_ (nm)	d_50_ (nm)	d_90_ (nm)	Concentration (Particle/mL)
1	142.7 ± 4.9	81.0 ± 2.8	77.5 ± 2.1	117.5 ± 3.2	231.2 ± 9.5	2.46 × 10^8^ ± 1.48 × 10^7^
2	198.5 ± 6.4	114.8 ± 11.8	94.8 ± 1.6	156.7 ± 8.6	253.5 ± 18.5	3.47 × 10^8^ ± 2.66 × 10^7^
3	187.1 ± 3.9	94.9 ± 3.0	91.5 ± 2.2	151.2 ± 2.8	261.3 ± 7.4	1.77 × 10^8^ ± 7.77 × 10^6^

**Table 4:** Lyosecretome total protein and lipid content; mean values ± standard deviation, *n* = 3. Different letters indicate a significant difference between groups (*p* < 0.0001).

Batch n.	μg Proteins/mg Lyosecretome	μg Lipids/mg Lyosecretome
1	10.0 ± 0.07 a	0.7 ± 0.05 a
2	13.0 ± 0.20 b	1.0 ± 0.04 b
3	10.6 ± 0.02 c	0.6 ± 0.07 c

## Data Availability

The data presented in this study are contained within the article.

## Supplementary Materials

The following are available online at https://www.mdpi.com/article/10.3390/ph14060553/s1, Figure S1. Functional evaluation of the protein profile characterized from equine mesenchymal stem/stromal cells. (A) Biological Processes, (B) Cellular Components, and (C) Molecular Functions enriched by considering protein secreted by equine mesenchymal stem/stromal cells. Fold enrichment was calculated by DAVID db (*p* < 0.01); Figure S2. Equus caballus protein–protein interaction (PPI) network reconstruction. (A) Homology between Equus caballus and Homo sapiens protein sequences. (B) Distribution of the global STRING score, (C) experiments STRING score, and (D) database STRING score found for the protein–protein interactions used to reconstruct the Equus caballus PPI network. Table S1. List of proteins identified by analyzing the Lyosecretome obtained by equine adipose-derived mesenchymal stem cells; Table S2. List of proteins involved in inflammatory processes and response to bacterium.
