# Supplementary material for: Equine Mesenchymal Stem/Stromal Cells Freeze-Dried Secretome (Lyosecretome) for the Treatment of Musculoskeletal Diseases: Production Process Validation and Batch Release Test for Clinical Use
Source: Pharmaceuticals (Basel). 2021 Jun 10;14(6):553. doi: 10.3390/ph14060553 (PMC8226765; doi:10.3390/ph14060553)
Supplement: Supplementary file 1 [file pharmaceuticals-14-00553-s001.zip › Supplementary materials.pdf]

Equine Lyosecretome for joint regeneration: production process validation and batch release test for clinical use

Michela Mocchi[1§], Stefano Grolli[2§], Silvia Dotti[3], Dario Di Silvestre[4], Riccardo Villa[3], Priscilla Berni[2], Virna Conti[2], Giulia Passignani[4], Francesca Brambilla[4], Laura Catenacci[1], Milena Sorrenti[1], Lorena Segale[5], Elia Bari[1], Pierluigi Mauri[4], Maria Luisa Torre[1,6*], and Sara Perteghella[1,6]

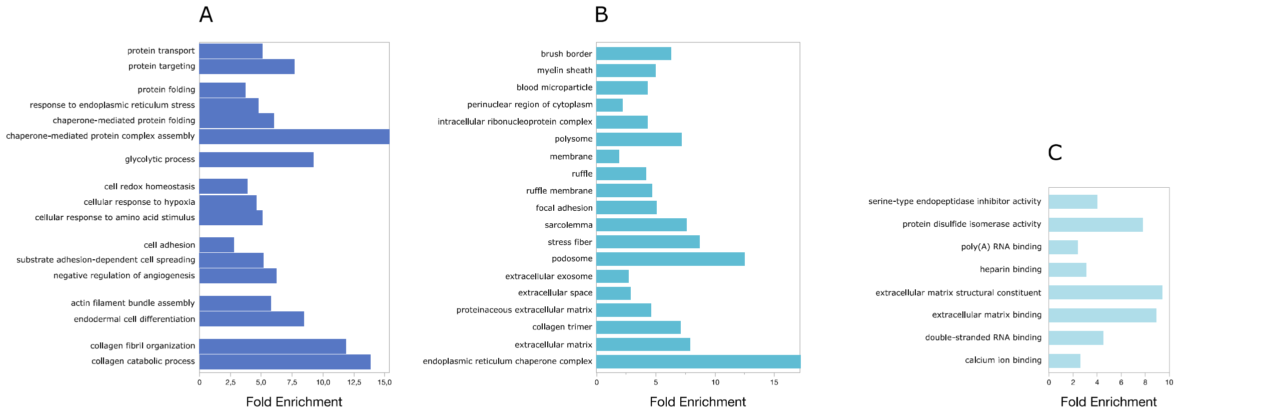

Figure S1. Functional evaluation of the protein profile characterized from equine mesenchymal stem/stromal cells. A) Biological Processes, B) Cellular Components and C) Molecular Functions enriched by considering protein secreted by equine mesenchymal stem/stromal cells. Fold enrichment was calculated by DAVID db ($p<0.01$).

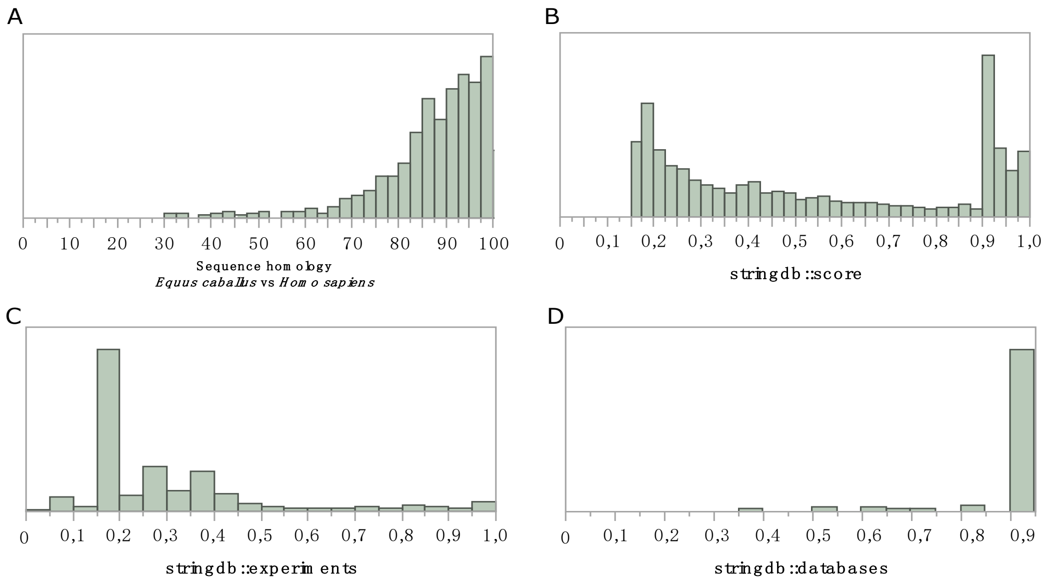

Figure S2. Equus caballus protein-protein interaction (PPI) network reconstruction. A) Homology between *Equus caballus* and *Homo sapiens* protein sequences. B) Distribution of the global STRING score, C) experiments STRING score and D) database STRING score found for the protein-protein interactions used to reconstruct the *Equus caballus* PPI network.
